# Electrical Coupling within Thalamocortical Networks Cumulatively Reduces Cortical Correlation to Sensory Inputs

**DOI:** 10.1523/ENEURO.0029-26.2026

**Published:** 2026-06-12

**Authors:** Austin J. Mendoza, Julie S. Haas

**Affiliations:** Department of Biological Sciences, Lehigh University, Bethlehem, Pennsylvania 18015

**Keywords:** cortex, electrical synapse, gap junction, thalamic reticular nucleus, thalamocortical, thalamus

## Abstract

Thalamocortical (TC) cells relay sensory information to the cortex as well as driving their own feedback inhibition through collateral excitation of the thalamic reticular nucleus (TRN). Inhibitory TRN cells are extensively coupled through electrical synapses. While electrical synapses are most often noted for synchronizing rhythmic forms of neuronal activity, their modulation of transient neuronal signals is less understood. Here we sought to characterize how electrical synapses embedded within a network of TRN neurons regulate the processing of ongoing sensory inputs during relay from thalamus to cortex. We constructed a thalamocortical network consisting of reciprocally connected Hodgkin–Huxley-style TC and TRN cells and one cortical output cell summing the TC activity. TRN cells were each electrically coupled to two neighboring cells, forming a ring topology. TC cells received synaptic inputs in sequence, with inputs separated by 10–50 ms, allowing us to assess the functional radius of an electrical synapse by comparing the cumulative effects of each additional TRN electrical synapse on responses within the network. Electrical synapse strength altered both TRN and TC spike response rates and latencies with each additional electrical synapse. Coupling within TRN modulated cortical integration of TC inputs by unexpectedly increasing response rates, duration, and reducing spike correlation to the input sequence that was presented to the TC layer. Thus, embedded TRN electrical synapses exert powerful influence on thalamocortical relay, highlighting the multisynaptic influences of electrically coupled cells on more complex and realistic networks of the brain.

## Significance Statement

Electrical synapses have been widely described as synchronizers, but their role in the processing of transient information flow has received less attention. In the thalamocortical system, electrical synapses couple cells of the reticular nucleus, which inhibit and modulate sensory transmission from thalamus to cortex. We used Hodgkin–Huxley-style computational models to investigate how electrical synapses embedded in a thalamocortical network impact the neural signals passing through it. Our models reveal accumulating effects across multiple electrical synapses that ultimately and unexpectedly diminish cortical correlation to an input sequence. Our results highlight the broad reach of electrical synapses.

## Introduction

Sensory information from the environment is communicated to the cortex by relay neurons of the thalamic nuclei. Rather than simple relay, however, it is generally agreed that neural information is transformed by thalamic circuitry. Ascending sensory information from all sensory systems, except for olfaction, targets cells of thalamic nuclei during transduction to higher brain regions (Sherman and Guillery, 2002; Jones, 2007). Thalamocortical (TC) cells within the primary sensory thalamic nuclei—the lateral geniculate nucleus for visual relay (Bickford, 2019), the ventral postmedial nucleus for touch (O'Reilly et al., 2021), and the medial geniculate body for auditory inputs (Bartlett, 2013)—receive strong “driver” inputs from sensory afferent pathways and send projections to corresponding cortical areas. Axons from TC cells also collateralize upon cells of the thalamic reticular nucleus (TRN) en route to the cortex (Scheibel and Scheibel, 1966; Lam and Sherman, 2011). The TRN is a shell of GABAergic neurons that surround the thalamus in its dorsal aspect (Pinault, 2004). Neurons of the TRN also receive excitation from corticothalamic cells of layer V and VI (Bourassa and Deschênes, 1995; Whilden et al., 2021; Carroll et al., 2022) and a wealth of modulatory input (McCormick and Wang, 1991; Pazo et al., 2013; Beierlein, 2014; Herrera et al., 2016; Aizenberg et al., 2019). In light of its potential to selectively inhibit sensory relay, TRN has been proposed to focus an attentional “searchlight” on behaviorally important information before it reaches the cortical input layer (Crick, 1984; McAlonan et al., 2008). A variety of behavioral and recording experiments have demonstrated the influence of TRN toward attentional tasks (McAlonan et al., 2000; McAlonan et al., 2006; Wimmer et al., 2015; Wells et al., 2016; Nakajima et al., 2019; Weese, 2022). Anatomical data also support potential intra-TRN and feedback connections that may implement the searchlight (Scheibel and Scheibel, 1966; Shosaku et al., 1989; Pinault et al., 1995; Pinault and Deschênes, 1998a,b). Yet the specific influences of TRN circuits on thalamocortical relay have not been well described.

Communication between TRN cells is proposed as a key feature of the hypothesized spotlight function in thalamocortical circuits (Pinault and Deschênes, 1998a). Whether the GABAergic neurons of the TRN chemically synapse onto each other remains controversial. Inhibitory responses have been recorded from glutamate uncaging experiments in juvenile mice (Deleuze and Huguenard, 2006; Lam et al., 2006), but optogenetic stimulation of ChR2-expressing TRN cells revealed that direct inhibitory synapses appear to be limited to earlier development (Hou et al., 2016). Only a single direct demonstration of a mixed inhibitory and electrical connection between paired TRN cells has been reported (Landisman and Coulon, 2024). Connectivity within TRN is instead mainly thought to rely on its dense population of electrical synapses (Landisman et al., 2002). Coupling between TRN cells appears to be extensive and may couple up to 20 cells with the average coupled network size being 9 cells, as revealed by dye-coupling experiments (Lee et al., 2014). Electrical synapses can play many diverse roles within networks; the type most often described and well understood is enhancement of synchrony among coupled neurons (Lewis and Rinzel, 2003; Christie et al., 2005; Leznik and Llinás, 2005; Pfeuty et al., 2005; Mancilla et al., 2007). TRN activities are more synchronous when electrical synapses are in a stronger state (Long et al., 2004). Beyond their synchronizing influence, recent large-scale simulations of TRN, which included electrically coupled networks, have often focused on aspects of rhythmicity or sleep spindle activity (Iavarone et al., 2023; Li et al., 2024). Electrical synapses of the TRN may exert other complex effects toward processing transient inputs; models have shown that the strength of coupling can merge closely timed inputs or act to further distinguish inputs with greater differences in timing or amplitude (Pham and Haas, 2018).

How electrical synapses impact the passage of transient inputs within TRN or across the brain's networks is not well understood. Previous demonstrations of transient signal processing by electrical synapses have focused on very local interactions. Coincident inputs received by two coupled cells can be enhanced through the presence of electrical synapses (Galarreta and Hestrin, 2001; Veruki and Hartveit, 2002). Similarly, temporally separated inputs to two neurons become weaker due to the current sink produced by a coupled quiescent cell (Trenholm et al., 2013; Alcami, 2018). Transmission of inhibitory afterhyperpolarizations by electrical synapses in a rhythmic context acts to desynchronize cell activity (Vervaeke et al., 2010; Hürkey et al., 2023). Coupling between cerebellar basket cells increases spiking probability in those cells through spikelet transmission, evoked within a short time window (Alcami, 2018; Hoehne et al., 2020). Similarly, electrical synapses enhance synchrony and also temporal precision in the toadfish vocal circuit (Chagnaud et al., 2021). Chemical EPSPs transmitted along a dendrite can be shunted by the presence of nearby electrical synapses (Llinás et al., 1974; Lang et al., 1996; Mendoza and Haas, 2022). These effects may also act to enhance signal-to-noise ratio in individual neurons through the combined effects described above (Ammer et al., 2022). In canonical feedforward circuits, electrical synapses between interneurons broaden the integration window of target cells and can enhance transmission efficiency in larger networks (Pham and Haas, 2019).

We sought to understand how electrical synapses embedded within a network of TC and TRN neurons regulate the processing of ongoing sensory inputs during relay to cortex. Spatially extending our previous model that used a single electrical synapse between two TRN neurons to reveal modulation of the temporal dynamics between two TC cells (Pham and Haas, 2018) before they are relayed to cortex, here we constructed a coupled network of a ring of nine TRN and nine TC cells receiving a sequence of synaptic inputs, such that each subsequent TC response is modulated by the prior responses of its neighbors. We also added a single cortical integrator neuron to the model in order to examine the ultimate impacts of TRN electrical synapses upon thalamocortical relay. This architecture allowed us to examine how synapses within TRN modulated responses of the TC cells and the information that is relayed to cortex. Our simulations showed us that spiking properties and, importantly, correlation and separation of spikes in the thalamocortical relay were altered as a function of coupling strength, highlighting the complex and pervasive roles of electrical synapses in ongoing activity as it passes through networks. These results also provide a basis for understanding how plasticity of electrical synapses might affect the networks in which they are embedded.

## Materials and Methods


$$\begin{array}{rl} C_{m}\frac{dV}{dt} & =I_{\mathrm{applied}}+G_{\mathrm{leak}}\cdot (E_{\mathrm{leak}}-V)\\ & \;+\underset{\mathrm{ion}\;\mathrm{channels}}{\sum }\;G_{\mathrm{ion}}\cdot (E_{\mathrm{ion}}-V)\\ & \;+\overset{n\neq i}{\underset{\mathrm{chemical}\;\mathrm{synapses}}{\sum }}\;G_{\mathrm{syn}}\cdot (E_{syn}-V)\\ & \;+\overset{n\neq i}{\underset{\mathrm{electrical}\;\mathrm{synapses}}{\sum }}\;G_{\mathrm{elec}}\cdot (V_{n}-V) \end{array}\}.$$

Equation (1) shows the Hodgkin–Huxley equation for model cells.

Models for all cells were single-point Hodgkin–Huxley models (Eq. 1), which we wrote in the Julia programing language and solved using the VCAB3 function from the DifferentialEquations.jl ODE solving suite with the third-order Adams method (Rackauckas and Nie, 2017). Simulations were run on an ASUS desktop PC with Intel i7-10700K CPU running Windows 11. Parameters for TC and TRN cells were built upon those previously reported (Destexhe et al., 1994; Traub et al., 2005; Pham and Haas, 2018) and included the following ionic currents and maximal conductances: fast transient Na+ (NaT) 60.5 mS/cm^2^, K+ delayed rectifier (Kd) 60 mS/cm^2^, K+ transient A (Kt) 5 mS/cm^2^, slowly inactivating K+ (K2) 0.5 mS/cm^2^, slow anomalous rectifier (AR) 0.025 mS/cm^2^, and low threshold transient Ca2+ (CaT) 0.75 mS/cm^2^. Reversal potentials were 50 mV for sodium, −100 mV for potassium, 125 mV for calcium, −40 mV for AR, and −75 mV for leak. Capacitance was 1 µF/cm^2^ with leak of 0.1 mS/cm^2^. Cortical output cell was a regular spiking neuron with only regular sodium and potassium currents and a leak of 0.1 mS/cm^2^ (Pospischil et al., 2008). Chemical synapses were modeled as double exponential decay with rise and fall time kinetics of 5 and 35 ms, respectively, with reversal potentials of 0 mV for excitatory and −100 mV for inhibitory synapses. We chose a conductance for synaptic inputs that, in the absence of other connections within the circuit, drove a four-spike burst in the TC neurons at a latency of ∼15 ms (Fig. 2*B*). We used excitatory chemical synapses between TC and TRN neurons strong enough to drive a three-spike burst in the TRN neurons at a latency of ∼30 ms from the input. Electrical synapses were modeled as static ohmic conductance applied to the voltage difference between the coupled TRN cells.

Scaling up to a network, neuronal excitability changes as coupling is increased between neurons (Amsalem et al., 2016). Thus, for each coupling strength, we systematically scaled TRN cell leak conductance so that the neuron's input resistance matched the input resistance of the uncoupled TRN model cell. As a result of this imposition, input resistances were close to constant for each cell in the network and thereby better linked changes in spiking to changes in electrical synapse strength. Sensory inputs were simulated as exponentially decaying current pulses (peak amplitude 0.75 µA/cm^2^, with decay time constant 30 ms) delivered to each of the TC cells. Chemical synaptic connections between TRN cells were set at 1 µA/cm^2^ for excitatory synapses from TC to TRN cells and 1 µA/cm^2^ for all inhibitory synapses originating from TRN. For each combination of stimulus timing and electrical synapse strength, we repeated 1,500 trials; each trial lasted 1.6 s, with arrival times of spontaneous synaptic inputs drawn from a Poisson distribution at a rate of 80 Hz for excitatory and 20 Hz for inhibitory events. Coupling also increased the baseline spontaneous firing rate of the TRN neurons; therefore, we scaled the amplitude of noise to maintain all cells at a spontaneous firing rate of 6–8 Hz across all simulations. This spontaneous rate was chosen to keep spontaneous spiking low enough to distinguish input evoked responses, while also similar to awake TRN firing (Marks and Roffwarg, 1993). Subthreshold current steps were applied to all cells to bring cells close to threshold, TC and TRN cells were depolarized to −70 mV, and cortical cells were given 0.3 µA/cm^2^. Spike train data was extracted and saved for further analysis and visualization using MATLAB.

### Spike train analysis

Separation of TC cell spikes were calculated as the time difference between the last spike of the first TC cell pair to receive the input and the first spike of the following cell response. Correlation was calculated from overlap of input time and spiking response time series, convolved with a falling exponential to produce similar time series to the delivered input. Latency was simply the difference between input onset and the time of the first spike in the response. Duration was the difference between the first and last spike of the response, responses consisting of only one spike were excluded from analysis. We calculated mean and standard errors of response variables across the 1,500 trials for a 100 ms time frame after input onset. Spike-triggered averages of synaptic currents were calculated from the time frame of 50 ms to 1 ms prior to the first spike of the response. Forward triggered averages were from the time frame of 1 ms to 50 ms after the first spike of the response. If no spike occurred within 100 ms following an input, the trial for that cell was excluded from calculated means and standard errors for those response characteristics.

### Code accessibility

The code/software described in the paper is freely available online at https://github.com/jhaaslab/RingModel. The code is available as Extended Data.

10.1523/ENEURO.0029-26.2026.d1 Data 1 Download Data 1, ZIP file.

## Results

**Figure 1.**
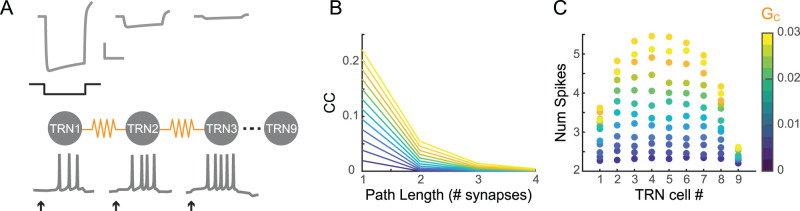
Effects of electrical synapses measured over network distance. ***A***, Simple network of identical coupled TRN neurons. Top traces, voltage responses to a hyperpolarizing current applied to TRN1. Bottom traces, spiking responses to identical excitatory inputs (arrows) delivered to each cell. ***B***, Coupling coefficients (cc) as a function of path length across the TRN network in ***A***, as measured from TRN1. ***C***, Average number of spikes in the response of each TRN cell in the network resulting from inputs shown in ***A***.

Our general goal was to investigate the reach of electrical synapses, that is, within a network that includes coupled neurons, the radius of their functional influence. First, we measured coupling coefficients between TRN cells using the commonly used experimental paradigm of delivering a hyperpolarizing pulse to one cell and measuring voltage deflections through the rest of the network (Fig. 1*A*, top). For the strongest synapses, a pulse that revealed a coupling coefficient of 0.2 in the neighboring cell diminished to 0.05 at the next cell and diminished to negligible thereafter (Fig. 1*B*). This sharp decay of coupling coefficients suggests that electrical synapses confer only minimal influence from one neuron onto neighbors more than two synapses away, even for strong electrical synapses. We then used the same network to compare responses to identical inputs to each of the neurons (Fig. 1*A*, bottom), with a delay of 20 ms between inputs. In this scenario, we noted that responses of each sequential cell in the chain included more and/or faster spikes, resulting in a cumulative rather than diminishing effect on spiking in each neighboring neuron (Fig. 1*C*). This exercise demonstrates that coupling coefficients may not capture or predict the impact of electrical synapses within active neuronal networks.

**Figure 2.**
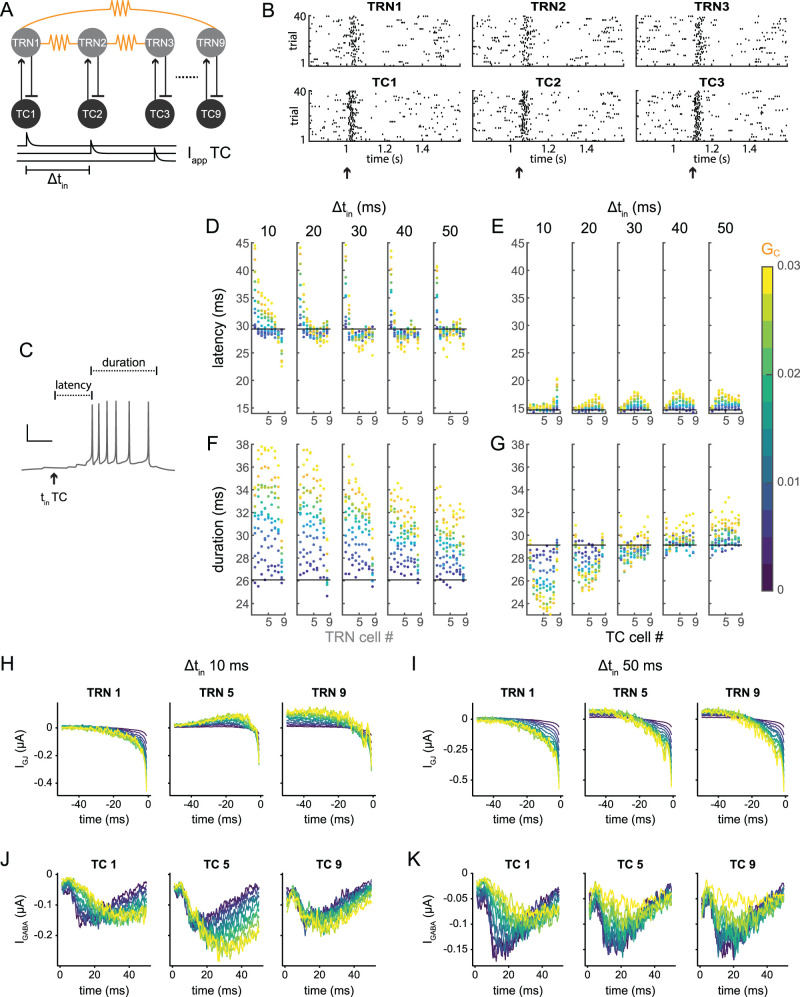
Coupling within the TRN layer modulates the latency and duration of TRN and TC spike trains. ***A***, Schematic of the ring network and inputs. ***B***, Example rasters of TRN and TC spike trains. ***C***, Representative trace showing measurements of spiking properties resulting from an input delivered to one TC cell. Scale bars: 25 mV, 20 ms. ***D***, Average spiking latency of TRN cells in the network with increasing *G*_c_. Black lines represent latency in an uncoupled network. Left to right panels, increasing Δ*t*_in_. ***E***, Average spiking latency of TC cells with increasing *G*_c_ within the TRN cell network. Black lines represent values in an uncoupled network. Left to right panels, increasing Δ*t*_in_. ***F***, Average spiking duration of TRN cells in the network with increasing *G*_c_ value. Black lines represent duration in an uncoupled network. Left to right panels, increasing Δ*t*_in_. ***G***, Average spiking duration of TC cells with increasing *G*_c_ within the TRN cell network. Black lines represent duration in an uncoupled control network. Left to right panels, increasing Δ*t*_in_. ***H***, Average gap junctional current (*I*_GJ_) preceding spikes (*t* = 0) in TRN cell 1 (left), TRN cell 5 (middle), TRN cell 9 (right) for Δ*t*_in_ = 10 ms across values of *G*_c_. ***I***, As in ***H***, for Δ*t*_in_ = 50 ms. ***J***, Average inhibitory current (*I*_GABA_) following a spike (*t* = 0) received by TC cell 1 (left), TC cell 5 (middle), TC cell 9 (right), for Δ*t*_in_ = 10 ms across values of *G*_c_. ***K***, As in ***J***, for Δ*t*_in_ = 50 ms.

We then set out to address how electrical synapses embedded within a network of TRN neurons regulate the processing of ongoing sensory inputs relayed by a layer of thalamocortical (TC) relay neurons. To use and understand a simplified thalamic network, we provided a sequence of synaptic inputs to the TC cells of a nine-cell ring (Fig. 2*A*) in which each TRN neuron was electrically coupled only to its closest neighbors, rather than forming the complex syncytium of the natural TRN. In this model, distance between coupled TRN neurons is synaptic rather than physical. Because coupling introduces a current sink to a neuron that reduces input resistance (Amsalem et al., 2016), we adjusted leak conductances current in order to maintain TRN input resistances at a constant value as we varied coupling across simulations. All cells were provided with spontaneous input such that they fired spontaneously at 6–8 Hz (Fig. 2*B*; Marks and Roffwarg, 1993); this choice rendered them more sensitive to the smaller synaptic inputs we provided, compared with neurons at rest that would require larger inputs. Synaptic inputs were delivered to TC cells at a fixed time difference (Δ*t*_in_), between 10 and 50 ms; we chose this set of inputs to represent a spectrum of thalamic inputs ranging from nearly identical to less similar (e.g., whiskers moving across a surface; similarly timed or frequencies of sound). These choices and input sequences gave us a basis for evaluating the impact of electrical synapses between TRN neurons, whose effects were exerted through the inhibitory synapses from each TRN cell to its paired TC cell, on thalamic relay of synaptic inputs.

To understand the impacts of electrical synapses within the TRN layer on TC spiking, we first measured the latency and duration of spiking responses in the TRN cells as a function of electrical synapse strength and input timing (Fig. 2*C*). In the TRN neurons, increases in electrical synapse strength increased the latency of TRN spiking (Fig. 2*D*), consistent with relative increases in the current sink in *I*_GJ_ imposed by stronger gap junctions to inactive neighbors. This effect was proportional to electrical synapse strength and was largest for TRN1 (Fig. 2*H*, left). Increased latency from the sink was countered by gap junction-mediated spread of input-driven excitation that accumulated in later TRN neurons (Fig. 2*H*, right). These results illustrate that by spreading either excitation of spiking or inhibition of a sink, electrical synapses bidirectionally amplify ongoing activity or its absence. Latency changes in TRN were largest for more closely timed inputs, where excitation from one input overlapped with arrival of the next input; for more separated inputs, the sink effect was larger (Fig. 2*I*). Duration of TRN spike bouts was also modulated by electrical synapses, with consistently longer duration proportional to coupling strength, as expected, from shared excitation between TRN neurons for shorter Δ*t*_in_. TRN duration diminished with increased Δ*t*_in_ (Fig. 2*F*), as shared excitation decayed more between bouts of spiking. Overall, effects of electrical synapses within TRN cells were strongest for the neurons closest to the initial input and diminish for the more distant cells, as expected.

Next, we turned to the TC layer. Because inputs arrived directly to TC cells first, latency of spiking was only minimally later in TC cells with greater TRN coupling strength (Fig. 2*E*). Duration of TC spiking (Fig. 2*G*) was strongly controlled by the inhibition delivered from TRN cells during a spiking bout (*I*_GABA_; Fig. 2*J*), which was in turn modulated by the strength of electrical synapses and shared excitation between TRN neurons. As a result, TC spike trains were truncated by up to 5 ms when both coupling was high between TRN cells and Δ*t*_in_ was small (Fig. 2*G*, left). We also noted a cumulative increase in the baseline tonic (*t* = 0) inhibitory currents from TC1 (0.015 µA) to TC9 (0.078 µA) for Δ*t*_in_ = 10 ms, resulting from cumulative shared activity across TRN neurons. For larger Δ*t*_in_, shorter duration of TRN bouts, domination with the TRN layer of the sink from gap junctions, and less overlap between inputs resulted in less inhibition delivered to TC neurons as electrical synapse strength increased (Fig. 2*K*), allowing the TC duration to increase progressively.

**Figure 3.**
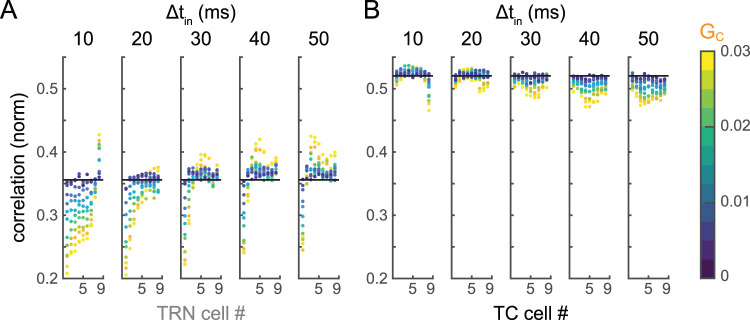
Electrical coupling decorrelates TRN and TC trains from sensory inputs. ***A***, Average correlation between input trains and TRN spiking for increasing *G*_c_ value. Black line represents correlation for an uncoupled network. Left to right panels, increasing Δ*t*_in_. ***B***, Average correlation between input trains and TC spiking for increasing *G*_c_ within the TRN layer. Black line represents separation correlation in an uncoupled network. Left to right panels, increasing Δ*t*_in_.

Electrical synapses in the TRN layer also modulated the correlations of TC and TRN neurons to the input. We measured correlation between the input times and spike trains in the coupled network, each convolved by a falling exponential with time constant 5 ms. TRN cells were least correlated to inputs for closely timed inputs and strong electrical synapses (Fig. 3*A*, left), diminishing by 50%, as a result of the GJ-driven changes in latency and duration of their spike trains described above. A cumulative effect of electrical synapses can be seen across TRN cells, producing a gradual increase in correlation from the first to last TRN to receive the input. Correlation of TC cells to their inputs was also modulated, although much less strongly, by GJs between the TRN neurons, and again in a cumulative manner across TC cells (Fig. 3*B*). These effects were driven mainly by changes in duration of TC spiking, which differed most greatly at the extremes of Δ*t*_in_ and produced the lowest correlations at those values as well.

**Figure 4.**
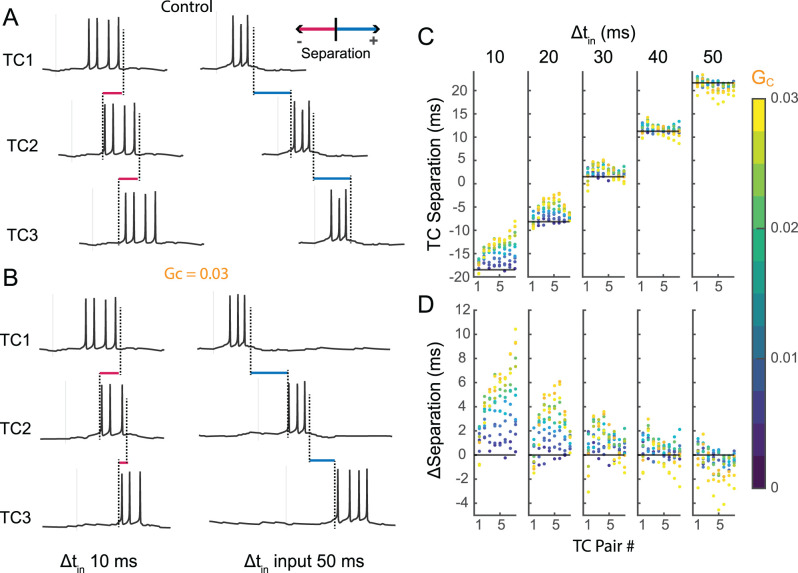
Coupling within TRN layer modulates separation of TC spike trains. ***A***, Representative traces from the first three TC cells for a control trial (no coupling between TRN cells). Left traces show separation with a small Δ*t*_in_, and right traces show separation with a large Δ*t*_in_, with separation notated. Horizontal dotted lines indicate the start and end of each spiking bout used to measure separation. ***B***, As in ***A***, for a trial with high coupling strength between TRN cells. ***C***, Average pairwise spike separations of TC cells in the network with increasing values of uniform *G*_c_ between TRN cells. Black lines represent separation in an uncoupled network. Left to right, increasing Δ*t*_in_. ***D***, Average pairwise changes in spike separation of TC cells, plotted for increasing *G_c_* within the TRN cell network, relative to the control response of TC to input alone (black lines). Panels show results for increasing Δ*t*_in_.

As a first step at understanding how cortical reception of thalamocortical relay is modulated by TRN electrical synapses, we measured the separation and overlap between TC spike train bouts (Fig. 4*A*; Pham and Haas, 2018). Separation, measured in ms, is the time gap between spike bouts and provides an indication of whether a postsynaptic neuron might be expected to discriminate between two bouts of spiking. Greater separation between TC spike trains potentially underlies discrimination by a cortical neuron that is integrating TC inputs. As a result of inhibition from the TRN spike trains, coupling strength altered the separation values of each pair of TC spike trains. When Δ*t*_in_ was small, TC spikes had significant overlap (separation < 0) in the uncoupled networks; increasing electrical synapse strength produced increased separation and reduced overlap (Fig. 4*B*, left). At longer Δ*t*_in_, where TC spikes were already positively separated, the overall separation in TC spike trains was reduced by greater electrical coupling (Fig. 4*B*, right). In both cases, the effects increased across pair number as more inputs were delivered to the TC layer, again demonstrating a cumulative effect due to network coupling (Fig. 4*C*). The magnitude of separation changes was considerable at the timescale of spike timing, as the effect of coupling produced up to 10 ms difference from control spike separations (Fig. 4*D*). In summary, the decorrelation of TC cell responses and thus changes in how separated responses were from control are produced by both latency and duration changes dependent on the coupling within the network. The consequences of these temporal changes predict substantial changes in how the cortex might integrate and process the arrival of these thalamocortical inputs.

**Figure 5.**
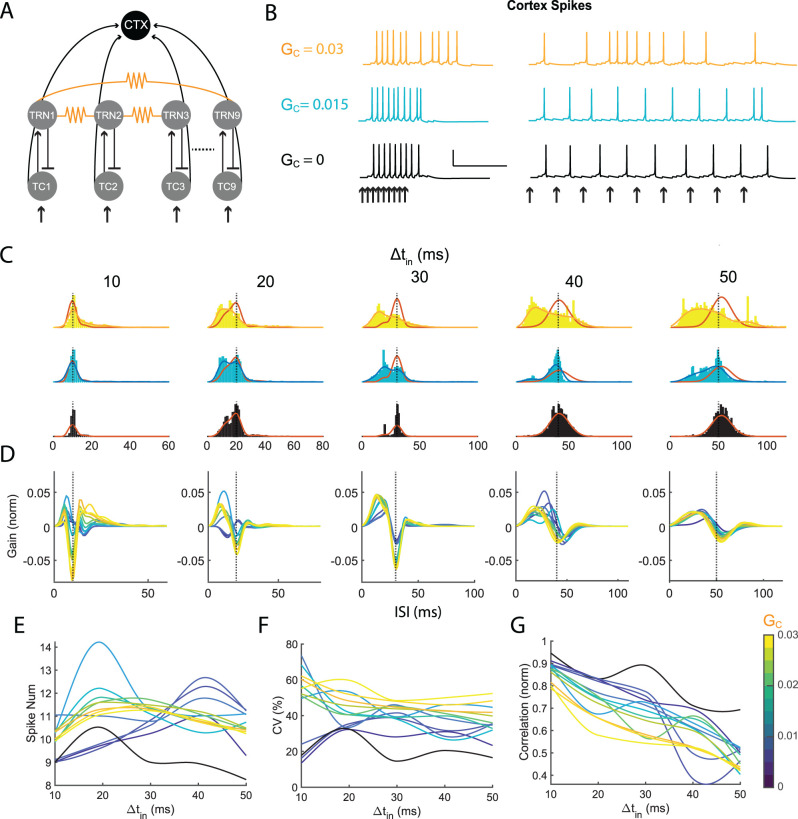
Impact of coupling within TRN on TC-relayed cortical spiking. ***A***, We added a cortical neuron to integrate TC relay from the ring network. ***B***, Cortical spike trains for control (no coupling between TRN cells), moderate (blue), and high (yellow) coupling between TRN cells within the network, for Δ*t*_in_ = 10 ms (left) and Δ*t*_in_ = 50 ms (right). ***C***, ISI distributions for the cortex cell for control (no coupling between TRN cells), moderate (blue), and high (yellow) coupling between TRN cells of the network. Mean ISI distributions for uncoupled TRN are overlayed in red. Left to right, increasing Δ*t*_in_. ***D***, Gain of the ISI distributions, versus time and across coupling values. ***E***, Average number of spikes in the cortex cell, versus Δ*t*_in_ and across coupling values. ***F***, Average coefficient of variation of the ISI distributions in ***C***, versus Δ*t*_in_ and across coupling values. ***G***, Average correlation between cortex spike times and input sequence times, versus Δ*t*_in_ and across coupling values.

Finally, we added a single simple integrator neuron to sum and read out the output of thalamocortical relay from our ring network (Fig. 5*A*). Because spontaneous TC spikes passed through TRN gap junctions could confound or mask the effects of electrical synapses passing input-driven TC spikes within the TRN layer on the ultimate cortical integration of those inputs, we ran these simulations in the absence of noise. Instead, to produce variation across trials, we delivered inputs with 5% variation in the input arrival times. We chose an excitatory synapse strength for TC inputs to the cortical neuron that produced a single spike in the cortical neuron for each TC input (Fig. 5*B*, bottom). Cortical spike times were nearly regular (CV of spike trains = 20–30%) for no or low TRN coupling. Surprisingly, we observed that increases in coupling within the TRN layer produced more spikes, irregular spikes within trains, and longer spike trains in the cortex cell across all Δ*t*_in_ values (Fig. 5*B*). Interspike interval (ISI) distributions for cortical spikes were tightly restricted around the Δ*t*_in_ value in the uncoupled and low-coupling ranges (Fig. 5*C*, bottom). With increased coupling strength within the TRN, ISI distributions in the cortical neuron broadened substantially (Fig. 5*C*, top). To better visualize this effect, we plotted the gain of the cortical ISI distributions in coupled cases relative to the ISI distributions for uncoupled TRN (Fig. 5*D*). These plots revealed a reduction in ISIs around the value of Δ*t*_in_ (dotted line) and a gain in shorter ISIs as coupling strength increased, indicative of the shift from regular spiking toward more irregular firing. Consistent with the increase in shorter ISIs, the average spiking output of the cortex cell increased for higher coupling within TRN (Fig. 5*E*). We quantified the effect on irregular firing by measuring the coefficient of variation (CV) of the ISI distribution. Spike trains in cortex were increased in CV to 60% at higher coupling values and across Δ*t*_in_ (Fig. 5*F*). Lastly, we asked if these spiking changes modulated the correlation of cortex spikes to the TC input train. Correlation in the cortex spikes to the input train was high (70–90%) for uncoupled and weakly coupled TRN and decreased (to 40–50%) as a result of increased coupling (Fig. 5*G*). We conclude that electrical synapses within the TRN have diverse impacts on TRN spiking, which accumulated and propagated to modulate TC cell responses, and ultimately, cortical responses to thalamocortical inputs.

## Discussion

While the role of electrical synapses in synchronizing rhythmic activity is well established, their contributions to the neuronal processing of transient inputs across networks remains less well characterized. Our computational investigation demonstrates that embedded electrical synapses have an unexpectedly decorrelating and potentially strong impact on thalamocortical relay that arises as the modulation of electrical synapses between TRN neurons propagates and accumulates through the network. Electrical synapses between TRN cells modulate latency and especially duration of TRN spike trains; these changes are passed into the TC cells through inhibitory synapses and impact the duration and correlation of TC spiking. The effects of each electrical synapse within the chain accumulate with each cell and result in dispersed and decorrelated responses in the cortical cell. These effects illustrate that TRN coupling influences activity through a large extended network, with effects on spiking output that would not be predicted from the passive measurements of each electrical synapse alone, as coupling between pairs alone would not predict the effects we see in a network context and drops to indistinguishable levels when measured by the traditional hyperpolarizing current steps across multiple synapses.

The TRN is hypothesized to focus a “searchlight” for information transmitted to cortex by thalamus (Crick, 1984; McAlonan et al., 2008), but the details of such a process remain underdescribed. Our results indicate that thalamocortical relay may be strongly modulated, and information transformed, by electrical coupling within TRN, as our results suggest ongoing activity is less faithfully transmitted when TRN cells are strongly coupled. We hypothesize that coupling might be modulated within small subnetworks of the TRN by activity or neuromodulators to selectively impact thalamocortical transmission. Numerous mechanisms of plasticity have been described in coupled systems (Lasater and Dowling, 1985; Pereda and Faber, 1996; Landisman and Connors, 2005; Haas et al., 2011; Mathy et al., 2014; Wang et al., 2015). Coupling across TRN could be temporarily upregulated across a sensory sector, or globally, in order to disrupt attention to the sensory surround, for instance. Alternatively, relay could be improved by weakened coupling in the relay channel for a specific object while increased coupling might disperse and interrupt relay in a neighboring channel, resulting in increased cortical reception of and attention to of the first channel. Many such schemes are possible. Though we did not include plasticity dynamics in our model, the effects of modifying synapse strengths within the network can be extrapolated from the synapse strengths we sampled in our network model to provide specific predictions.

TRN cells couple small clusters of nearby cells with direct electrical synapses; our model here considers a specific topology of connections in order to address the extent of electrical synapse influence over spike times. Effects of gap junctions on transient inputs, which note excitatory effects for closely timed inputs, would also propagate across several cells in a network context and have considerable impacts on information processing (Galarreta and Hestrin, 2001; Vervaeke et al., 2010; Trenholm et al., 2013; Pham and Haas, 2018; Hoehne et al., 2020). Our simulations provide basic investigations into larger electrical synaptic embedded networks at TRN, which can form a basis of comparison for future investigations. In particular, whether these results can be reproduced in a more naturalistic topology in which connections are randomly dispersed among nearby cells that form a syncytium remains to be determined. In live tissue, electrical synapses are stronger and more numerous for cells with closer intersomatic distances (Long et al., 2004). The results here, where cortical integration is influenced by alterations in TC firing resulting from TRN coupling, may vary in vivo. We expect that impacts on cortical integration would be strongest for nearby TC cells if the TRN cells they receive inputs from are highly coupled. TC cells may receive input from multiple coupled TRN neurons, possibly even amplifying these effects.

Many refinements are possible for our network model that could impact its predictions. Our thalamic cell models were detailed in order to include the important bursting behaviors in those cell types (McCormick and Huguenard, 1992; Destexhe et al., 1996; Destexhe et al., 1998), but we opted for a simplistic cortical output neuron so that changes we observed in responses were due to the electrical synapse-mediated changes in the relay alone. We expect that intrinsic properties of cortical cells could be another important source of modulation in cortical response that would interact with the modulation by TRN synapses. Thus, further study could include more detailed neuron models for layer 4 and include specifics such as spike adaptation due to M-currents or dendritic morphology and dynamics. Further, deep-layer cortical neurons provide an important source of input to TRN (Gentet and Ulrich, 2004; Whilden et al., 2021; Carroll et al., 2022; Hádinger et al., 2023; Sokhadze et al., 2025), and the impacts of that connection combined with electrical synapses within TRN should also be expected to modulate thalamocortical transmission. Also absent from our models are lateral inhibitory connections between nearby TRN cells (Deleuze and Huguenard, 2006; Lam et al., 2006; Landisman and Coulon, 2024). Corticothalamic feedback onto both TC and TRN neurons, although delayed, could be another avenue of investigation for future experiments. Another key thalamic synapse proposed as a mechanism of the searchlight is open-loop divergent inhibition from TRN to TC cells (Crabtree et al., 1998; Crabtree and Isaac, 2002).

TRN has recently been shown to comprise distinct cell types which differentially target thalamic relays of first and higher-order information (Clemente-Perez et al., 2017; Martinez-Garcia et al., 2020). Our results here only consider one homogenous source of TC relay and do not include the influence of these cell types and the potential sensory modulation that higher-order sources may contribute (Bu et al., 2025). Electrical synapses have been shown to couple TRN cells across cell types (Vaughn et al., 2024); additionally, somatostatin-expressing TRN may provide lateral inhibition to the first-order parvalbumin-expressing TRN cell type (Rolón-Martínez et al., 2026). In all, our model can provide important predictions for the diverse connections both within and between thalamic sources and their impact on precise sensory relay to the cortex.

## Synthesis

Reviewing Editor: Arianna Maffei, Stony Brook University Decisions are customarily a result of the Reviewing Editor and the peer reviewers coming together and discussing their recommendations until a consensus is reached. When revisions are invited, a fact-based synthesis statement explaining their decision and outlining what is needed to prepare a revision will be listed below. The following reviewer(s) agreed to reveal their identity: Gabrielle Gutierrez.

The reviewers found the study interesting and potentially impactful. They both identified points in the manuscript that need clarifications. Reviewer #1 raise the issue that the study extends a previously published study by Pham and Haas and requested to include clarifications for how this manuscript differs from previous work.

Reviewer #2 raised some concerns about details included in the model.

Specific comments are copied below.

Reviewer #1

Line 153 - This sentence is unclear. Please rephrase for clarity.

Line 228 - I'm a bit dubious about the reason for introducing variability into the inputs instead of with spontaneous spiking as before. It seems like circular reasoning to say that spontaneous TC spikes could "mask or confound the effects of electrical synapses" since your study is meant to investigate the effects of electrical synapses and presumably, you don't know what those effects will be a priori. Perhaps these doubts can be mitigated by including a supplementary figure that shows your results when variability is implemented through the same spontaneous spiking mechanism as before. At the very least, a more rigorous explanation for this choice should be written into this part of the Results.

Line 266 - This paragraph discusses network topology and plasticity, but it doesn't relate those two things. I think this should be split into 2 paragraphs, or the relationship needs to be made clearer.

Line 310 - This sentence is unclear. Please rephrase for clarity.

Line 311 - More detail about the integration method used by the solver is needed since these details have an impact on the results. Was it Runge-Kutte? Euler?

Line 326 - Scaling down the leak conductance to maintain a consistent input resistance is reasonable to do if there is evidence that TRN cells generally have a consistent input resistance regardless of their electrical coupling strengths. Has the input resistance of TRN cells been measured? If so, how variable are they? This modeling choice would be stronger if supported by biological measurements. Otherwise, this part should state that you have made an assumption here.

Line 331 - Please be explicit about which variable Poisson noise was applied to.

Line 332 - Scaling the amplitude of the noise to maintain a consistent firing rate is another modeling choice that should be justified. Do measurements of TRNs show that they are typically firing at between 6-8 Hz?

Line 341 - replace "convoluted" with "convolved with".

Line 345 - Were there 1500 trials in the whole paper, or is that 1500 trials for each condition? How long was each trial? More specificity is needed in this paragraph about your analyses.

Reviewer #2

Major comments:

1.) A major caveat of the model network presented in this manuscript is that the gap junction connectivity deviates substantially from the known anatomy of the TRN and other coupled neurons. Specifically, in this study, there are 9 TRN neurons with each TRN neuron coupled to its neighbor and the 9th neuron coupled back to 1st neuron. This topology ignores a fundamental aspect of gap junction connectivity, which is the distance-dependence of coupling conductance. Coupled networks form a continuous syncytium of gap junction connectivity, in which coupling conductance and density (both in cortex and TRN) is known to be strongest for nearby (<100 μm) neurons, with conductance and density decreasing with intersomatic distance, becoming negligible by ~200 μm.

The authors' model does not seem to recapitulate this fundamental characteristic of gap junction connectivity. If the positions of the neurons in the authors' ring topology correspond to points in 2D space, then something like the following should be the case to model the actual connectivity structure of TRN: TRN1 is coupled with equal (high) probability and strength to TRN2 and TRN9, TRN 1 is coupled with equal (lower) probability and strength to TRN3 and TRN8, and so one for TRN 4 and 7 and TRN 5 and 6. The authors need to comment more on the departure of their model from actual gap junction connectivity in TRN. Currently, their caveat about this issue is limited to only one sentence in the Discussion (lines 273-275). It would also be helpful for the authors to offer predictions about how the results would play out if the model followed more realistic topology. Would we see results that were completely different from the current simulations (i.e. stronger correlating actions of TRN electrical synapses) or would the decorrelating actions be expected to be even more extreme (i.e. even stronger reductions in TC spiking duration and even more irregular firing in the cortical integrator).

2.) I found mechanistic insight into the model's behavior to be lacking. One of the benefits of computational models (especially conductance-based models like the one the authors use) is that (unlike in many experimental preparations) we have complete access to everything that gives rise to the higher-level features of the simulations (i.e. spiking activity). Here, however, the authors only report spiking activity. We have no details about the subthreshold events (especially the electrical postsynaptic potentials ePSPs) or the excitatory or inhibitory chemical conductances that lead to the ultimate changes in neuronal spiking affected by varying the TRN coupling conductances. Take, as an example, the results in Figure 2D. We see that TRN9's spike latency for Δt = 10 ms actually decreases as coupling conductance increases. This is presumably because the subthreshold membrane potential was higher just prior to their TC synaptic input due to the temporal summation of ePSPs that were received from TRNs 1-8 when they received earlier synaptic input from the TC layer. Showing spike-triggered membrane potential would clarify the mechanisms behind the spiking behavior of both TRN and TC neurons at different positions in the network. Also, how do the TRN-coupling-dependent results observed in TC neurons (such as their lower spiking duration, longer latency, and reduced correlations) relate to the characteristics of the inhibition they receive from TRN? Does increased coupling conductance in TRN lead to larger chemical synaptic inhibitory conductances in TC neurons? If so, is this an overall, DC-level of tonic inhibition or is the inhibition phasic in character (with upward deflections in inhibitory conductance that arise out of a low baseline conductance). Presumably, it's the former (especially if TRN spike correlations are decreased with coupling, though this isn't shown [see more below]). Could it be that (counterintuitively) increased TRN coupling leads to lower phasic inhibitory input to TC neurons thereby causing decorrelation in their spiking? This would be an interesting result, and one that the authors have access to in their simulations, but the readers currently have no access to anything other than results for the spiking data.

Minor comments:

1.) The temporal organization of the "sensory" synaptic inputs to the TC neurons in the model seems not to match their topographic organization. Specifically, when the authors stimulate the TC layer, TC1 is stimulated first, then TC2, and so on until TC9 is stimulated last. But if TC1 is equidistant to TC2 and TC9 (as a consequence of the ring topology), shouldn't TC2 and TC9 receive temporally aligned synaptic input to reflect this?

2.) Lines 180-182: "In the TRN neurons, increases in electrical synapse strength increased the latency of TRN spiking (Figure 2D), consistent with increases in leak conductance." This statement is confusing because it was stated earlier (lines 172-175) that leak conductances were altered to account for gap junction conductances in order to maintain constant input resistance. This would presumably mean that an increase in coupling conductance was accompanied by an imposed decrease in leak conductance. Please clarify.

3.) Lines 186-187: "Overall, effects of electrical synapses within TRN cells were strongest for the several neurons closest to each synapse, as is intuitive." This statement is unclear because every TRN cell has an electrical synapse, so how can the effect be "strongest" for the "several neurons closest to each synapse." Please clarify.

4.) The computation depicted by Figure 3a is indecipherable to me. This figure presents data on the correlation between convolved TC spike trains with the synaptic inputs given to the whole TC input layer. Figure 3a does not really indicate this to me. You show two spike trains, which makes it seem like the two spike trains are being correlated. The very bottom of Figure 3a also makes no sense to me. What is the shorter purple line superimposed on the longer black line supposed to indicate? What about the dotted bracket labeled "correlation." There must be a better way to indicate the correlation computation. If not, I suggest taking the schematic out completely and just rely on the textual description.

5.) Lines 189-191: "Those increases were a result of increased spontaneous activity within the TRN layer mediated by increased GJ strength; that activity then resulted in increased inhibition delivered to TC cells." These properties are asserted, but not shown. As the reader, we don't see any quantified data on spontaneous TRN firing rate or increased inhibition received by TC neurons. If the authors are making causal statements about how gap junction coupling is affecting network dynamics, these statements need to be backed up with simulation data.

6.) In Fig. 4 a,b, increased GJ conductance between TRNs seems to increase separation between spiking *bouts* in TCs (due to the decreased spiking duration), but what about spike synchrony? That is, it could be that spiking *bouts* are more separated, but that tight (~1-3 ms or less) spike synchrony (as measured, e.g., by spike-count correlograms) actually increases. Also, data are shown for spike separation in TC neurons, but what about for TRN neurons. Do they show the same behavior? What about tight spike synchrony in TRN neurons? Does this synchrony counterintuitively diminish with increased TRN gap junction strength? If so, can this be traced to subthreshold ePSP dynamics and/or excitatory conductances from TC neurons?

7.) I don't understand the "separation" metric used in Figure 4. Presumably this is computed on spiking *bouts* of two neurons, and not temporal overlap of individual spikes from those in neurons? That should be made clear. I also can't understand whether "separation" is a bounded measure, like an index. It seems that the more positive the "separation" value is, the more distant in time two spiking bouts are. But what about the more negative "separation" values? It looks like a larger negative value indicates more overlap in spiking bouts, but suppose two spiking bouts overlapped completely. What "separation" value would they have? Presumably a very negative value, but if the "separation" value isn't bounded like an index, how are we to interpret the value that would correspond to the case of maximal spike bout overlap? Relatedly, what does "separation" value of 0 indicate? Does that mean a bout in one cell happens immediately after another? Why not just calculate this as percent overlap in time of any two spike bouts? I don't understand what this "separation" value lends us.

## Author Response

Synthesis of Reviews:

Computational Neuroscience Model Code Accessibility Comments for Author (Required):

N/A Synthesis Statement for Author (Required):

The reviewers found the study interesting and potentially impactful. They both identified points in the manuscript that need clarifications.

To both reviewers: Thank you for your time and expert attention. We genuinely appreciate the comments and opportunity to improve especially the clarity of our work.

Reviewer #1 raise the issue that the study extends a previously published study by Pham and Haas and requested to include clarifications for how this manuscript differs from previous work.

We revised the Introduction to clarify our extension of Pham and Haas (changes underlined here): "Spatially extending the previous model that used a single electrical synapse between two TRN neurons to reveal modulation of the temporal dynamics between two TC cells (Pham and Haas, 2018) before they are relayed to cortex, here we constructed a coupled network of a ring of 9 TRN and 9 TC cells receiving a sequence of synaptic inputs, such that each subsequent TC response is modulated by the prior responses of its neighbors. We also added a single cortical integrator neuron to the model in order to examine the ultimate impacts of TRN electrical synapses upon thalamocortical relay." Reviewer #2 raised some concerns about details included in the model.

Specific comments are copied below.

Reviewer #1 Line 153 - This sentence is unclear. Please rephrase for clarity.

We edited this sentence to read "This sharp decay of coupling coefficients suggests that electrical synapses confer only minimal influence from one neuron onto neighbors more than two synapses away, even for strong electrical synapses." Line 228 - I'm a bit dubious about the reason for introducing variability into the inputs instead of with spontaneous spiking as before. It seems like circular reasoning to say that spontaneous TC spikes could "mask or confound the effects of electrical synapses" since your study is meant to investigate the effects of electrical synapses and presumably, you don't know what those effects will be a priori. Perhaps these doubts can be mitigated by including a supplementary figure that shows your results when variability is implemented through the same spontaneous spiking mechanism as before. At the very least, a more rigorous explanation for this choice should be written into this part of the Results.

We modified the text here to make our intention clearer, which was to focus only on input passing through the network: "Because spontaneous TC spikes passed through TRN gap junctions could confound or mask the effects of electrical synapses passing input-driven TC spikes within the TRN layer on cortical integration of those inputs, we ran these simulations in the absence of noise." Line 266 - This paragraph discusses network topology and plasticity, but it doesn't relate those two things. I think this should be split into 2 paragraphs, or the relationship needs to be made clearer.

We agree, and we split these ideas into two paragraphs to distinguish them.

Line 310 - This sentence is unclear. Please rephrase for clarity.

We edited this to "Models for all cells were single-point Hodgkin-Huxley models (Eq. 1), which we wrote in the Julia programing language and solved using the VCAB3 function from the DifferentialEquations.jl ODE solving suite with the 3rd order Adams method (Rackauckas and Nie, 2017)." Line 311 - More detail about the integration method used by the solver is needed since these details have an impact on the results. Was it Runge-Kutte? Euler? Solver function and details have been added to the sentence above.

Line 326 - Scaling down the leak conductance to maintain a consistent input resistance is reasonable to do if there is evidence that TRN cells generally have a consistent input resistance regardless of their electrical coupling strengths. Has the input resistance of TRN cells been measured? If so, how variable are they? This modeling choice would be stronger if supported by biological measurements. Otherwise, this part should state that you have made an assumption here.

TRN cells do have a wide range of input resistances, from several tens to several hundred MOhms, and the relationship between input resistance and coupling strength is complex. Here, though, instead of sampling a broad distribution we wanted the excitability of each TRN cell to be ~constant across coupling strengths, so that timing effects would reflect coupling rather than increased excitability.

To be more specific about what we did, we changed the text to "Scaling up to a network, neuronal excitability changes as coupling is increased between neurons (Amsalem et al., 2016). Thus, for each coupling strength, we systematically scaled TRN cell leak conductance so that the neuron's input resistance matched the input resistance of the uncoupled TRN model cell. As a result of this imposition, input resistances was close to constant for each cell in the network, and thereby better linked changes in spiking to changes in electrical synapse strengths." Line 331 - Please be explicit about which variable Poisson noise was applied to.

We now specify that arrival times of spontaneous excitatory and inhibitory inputs were drawn from Poisson distributions.

Line 332 - Scaling the amplitude of the noise to maintain a consistent firing rate is another modeling choice that should be justified. Do measurements of TRNs show that they are typically firing at between 6-8 Hz? TRN neurons fire up to 40 Hz during awake states in vivo in moving rats but decreases to < 10 without movement (Marks and Roffwarg, 1993); we added this citation to the text.

Line 341 - replace "convoluted" with "convolved with".

Done, thank you for noticing this typo that Word did not! Line 345 - Were there 1500 trials in the whole paper, or is that 1500 trials for each condition? How long was each trial? More specificity is needed in this paragraph about your analyses.

This sentence now reads "For each combination of stimulus timing and electrical synapse strength, we repeated 1500 trials; each trial lasted 1.6 seconds, with arrival times of spontaneous synaptic inputs drawn from a Poisson distribution at a rate of 80 Hz for excitatory and 20 Hz for inhibitory events." Reviewer #2 Major comments:

1.) A major caveat of the model network presented in this manuscript is that the gap junction connectivity deviates substantially from the known anatomy of the TRN and other coupled neurons. Specifically, in this study, there are 9 TRN neurons with each TRN neuron coupled to its neighbor and the 9th neuron coupled back to 1st neuron. This topology ignores a fundamental aspect of gap junction connectivity, which is the distance-dependence of coupling conductance. Coupled networks form a continuous syncytium of gap junction connectivity, in which coupling conductance and density (both in cortex and TRN) is known to be strongest for nearby (<100 μm) neurons, with conductance and density decreasing with intersomatic distance, becoming negligible by ~200 μm.

The authors' model does not seem to recapitulate this fundamental characteristic of gap junction connectivity. If the positions of the neurons in the authors' ring topology correspond to points in 2D space, then something like the following should be the case to model the actual connectivity structure of TRN: TRN1 is coupled with equal (high) probability and strength to TRN2 and TRN9, TRN 1 is coupled with equal (lower) probability and strength to TRN3 and TRN8, and so one for TRN 4 and 7 and TRN 5 and 6. The authors need to comment more on the departure of their model from actual gap junction connectivity in TRN. Currently, their caveat about this issue is limited to only one sentence in the Discussion (lines 273-275). It would also be helpful for the authors to offer predictions about how the results would play out if the model followed more realistic topology. Would we see results that were completely different from the current simulations (i.e. stronger correlating actions of TRN electrical synapses) or would the decorrelating actions be expected to be even more extreme (i.e. even stronger reductions in TC spiking duration and even more irregular firing in the cortical integrator).

We agree that coupling strength and incidence have been shown to be distance dependent, in terms of microns separating pairs of cell bodies in experiments. However, the distance in our work is electrical, or synaptic distance, not physical space. We also simplified the numbers and geometry of connections to make our simulations and its results more tractable; we agree that a real network would more closely resemble some sort of randomized syncytium. To clarify these points, we added text "where each TRN neuron was coupled only to its closest neighbors rather than forming the complex syncytium of the natural TRN. In this model, distance between coupled TRN neurons is synaptic rather than physical." to the Results where the model is first described.

We have also added text to the discussion to speculate on the influence of topology on responses of live TC neurons (near line 305): "In live tissue, electrical synapses are stronger and more numerous for cells with closer intersomatic distances (Long et al. 2004). The results here, where cortical integration is influenced by alterations in TC firing resulting from TRN coupling, may vary in vivo. We expect that impacts on cortical integration would be strongest for nearby TC cells if the TRN cells they receive inputs from are highly coupled. TC cells may receive input from multiple coupled TRN neurons, possibly even amplifying these effects." 2.) I found mechanistic insight into the model's behavior to be lacking. One of the benefits of computational models (especially conductance-based models like the one the authors use) is that (unlike in many experimental preparations) we have complete access to everything that gives rise to the higher-level features of the simulations (i.e. spiking activity). Here, however, the authors only report spiking activity. We have no details about the subthreshold events (especially the electrical postsynaptic potentials ePSPs) or the excitatory or inhibitory chemical conductances that lead to the ultimate changes in neuronal spiking affected by varying the TRN coupling conductances. Take, as an example, the results in Figure 2D. We see that TRN9's spike latency for Δt = 10 ms actually decreases as coupling conductance increases. This is presumably because the subthreshold membrane potential was higher just prior to their TC synaptic input due to the temporal summation of ePSPs that were received from TRNs 1-8 when they received earlier synaptic input from the TC layer. Showing spike-triggered membrane potential would clarify the mechanisms behind the spiking behavior of both TRN and TC neurons at different positions in the network.

Also, how do the TRN-coupling-dependent results observed in TC neurons (such as their lower spiking duration, longer latency, and reduced correlations) relate to the characteristics of the inhibition they receive from TRN? Does increased coupling conductance in TRN lead to larger chemical synaptic inhibitory conductances in TC neurons? If so, is this an overall, DC-level of tonic inhibition or is the inhibition phasic in character (with upward deflections in inhibitory conductance that arise out of a low baseline conductance). Presumably, it's the former (especially if TRN spike correlations are decreased with coupling, though this isn't shown [see more below]). Could it be that (counterintuitively) increased TRN coupling leads to lower phasic inhibitory input to TC neurons thereby causing decorrelation in their spiking? This would be an interesting result, and one that the authors have access to in their simulations, but the readers currently have no access to anything other than results for the spiking data.

Thank you for this truly excellent suggestion. We have added panels to Figure 2 to show the spike triggered averages of the membrane currents that underlie the differences in spiking in both sets of cells.

Figure 2 H and I show the summed gap junctional currents preceding spikes in TRN cells as a function of GJ strength. Figures 2 J and K show the summed inhibitory currents following spikes that control duration of TC spiking. Both sets of currents are modulated by gap junction strength. We added the following text to explain changes in latency and duration in both sets of neurons in detail as a function of these synaptic currents within the network: "In the TRN neurons, increases in electrical synapse strength increased the latency of TRN spiking (Figure 2D), consistent with relative increases in the current sink in IGJ imposed by stronger gap junctions to inactive neighbors. This effect was proportional to electrical synapse strength and was largest for TRN1 (Figure 2H, left). Increased latency from the sink was countered by gap junction-mediated spread of input-driven excitation that accumulated in later TRN neurons (Figure 2H, right). These results illustrate that by spreading either excitation of spiking or inhibition of a sink, electrical synapses bidirectionally amplify ongoing activity or its absence. Latency changes in TRN were largest for more closely timed inputs, where excitation from one input overlapped with arrival of the next input; for more separated inputs, the sink effect was larger (Figure 2I). Duration of TRN spike bouts was also modulated by electrical synapses, with consistently longer duration proportional to coupling strength, as expected, from shared excitation between TRN neurons for shorter Δtin. TRN duration diminished with increased Δtin (Figure 2F), as shared excitation decayed more between bouts of spiking. Overall, effects of electrical synapses within TRN cells were strongest for the neurons closest to the initial input and diminishes for the more distant cells, as expected.

Next, we turned to the TC layer. Because inputs arrived directly to TC cells first, latency of spiking was only minimally later in TC cells with greater TRN coupling strength (Figure 2E). Duration of TC spiking (Figure 2G) was strongly controlled by the inhibition delivered from TRN cells during a spiking bout (IGABA; Figure 2J), which was in turn modulated by the strength of electrical synapses and shared excitation between TRN neurons. As a result, TC spike trains were truncated by up to 5 ms when both coupling was high between TRN cells and Δtin was small (Figure 2G, left). We also noted a cumulative increase in the baseline tonic (t = 0) inhibitory currents from TC1 (0.015 µA) to TC9 (0.078 µA) for Δtin = 10 ms, resulting from cumulative shared activity across TRN neurons. For larger Δtin, shorter duration of TRN bouts, domination with the TRN layer of the sink from gap junctions, and less overlap between inputs resulted in less inhibition delivered to TC neurons as electrical synapse strength increased (Figure 2K), allowing the TC duration to increase progressively." Minor comments:

1.) The temporal organization of the "sensory" synaptic inputs to the TC neurons in the model seems not to match their topographic organization. Specifically, when the authors stimulate the TC layer, TC1 is stimulated first, then TC2, and so on until TC9 is stimulated last. But if TC1 is equidistant to TC2 and TC9 (as a consequence of the ring topology), shouldn't TC2 and TC9 receive temporally aligned synaptic input to reflect this? This was an option we considered; in that case we would expect one half of the circle to mirror the other half, and inputs would collide sooner. To study inputs and their effects without collisions, we isolated work to a single sequence of inputs.

2.) Lines 180-182: "In the TRN neurons, increases in electrical synapse strength increased the latency of TRN spiking (Figure 2D), consistent with increases in leak conductance." This statement is confusing because it was stated earlier (lines 172-175) that leak conductances were altered to account for gap junction conductances in order to maintain constant input resistance. This would presumably mean that an increase in coupling conductance was accompanied by an imposed decrease in leak conductance. Please clarify.

We rewrote this as "In the TRN neurons, increases in electrical synapse strength increased the latency of TRN spiking (Figure 2D), consistent with relative increases in current sink imposed by stronger gap junctions." 3.) Lines 186-187: "Overall, effects of electrical synapses within TRN cells were strongest for the several neurons closest to each synapse, as is intuitive." This statement is unclear because every TRN cell has an electrical synapse, so how can the effect be "strongest" for the "several neurons closest to each synapse." Please clarify.

We rewrote this as "Overall, effects of electrical synapses within TRN cells were strongest for the neurons closest to the initial input and diminishes for the more distant cells, as expected." 4.) The computation depicted by Figure 3a is indecipherable to me. This figure presents data on the correlation between convolved TC spike trains with the synaptic inputs given to the whole TC input layer. Figure 3a does not really indicate this to me. You show two spike trains, which makes it seem like the two spike trains are being correlated. The very bottom of Figure 3a also makes no sense to me. What is the shorter purple line superimposed on the longer black line supposed to indicate? What about the dotted bracket labeled "correlation." There must be a better way to indicate the correlation computation. If not, I suggest taking the schematic out completely and just rely on the textual description.

We apologize for the lack of clarity; we have removed the schematic as suggested.

5.) Lines 189-191: "Those increases were a result of increased spontaneous activity within the TRN layer mediated by increased GJ strength; that activity then resulted in increased inhibition delivered to TC cells." These properties are asserted, but not shown. As the reader, we don't see any quantified data on spontaneous TRN firing rate or increased inhibition received by TC neurons. If the authors are making causal statements about how gap junction coupling is affecting network dynamics, these statements need to be backed up with simulation data.

Thank you for pointing out that this statement was not properly backed up by the results; we have removed this statement. We hope this issue is now clearer within the revised text.

6.) In Fig. 4 a,b, increased GJ conductance between TRNs seems to increase separation between spiking bouts in TCs (due to the decreased spiking duration), but what about spike synchrony? That is, it could be that spiking bouts are more separated, but that tight (~1-3 ms or less) spike synchrony (as measured, e.g., by spike-count correlograms) actually increases. Also, data are shown for spike separation in TC neurons, but what about for TRN neurons. Do they show the same behavior? What about tight spike synchrony in TRN neurons? Does this synchrony counterintuitively diminish with increased TRN gap junction strength? If so, can this be traced to subthreshold ePSP dynamics and/or excitatory conductances from TC neurons? We appreciate the focus on synchronized spiking, especially at fine timescales. However, we'd like to point out that the design choice for our work was to consider transient inputs, rather than synchronous inputs, to and within the thalamocortical network. Because each TC-TRN pair receives inputs in a sequence rather than synchronously, TC spikes in each cell are driven by non-synchronous inputs and modulated by different TRN cells in each case. Due to the asynchronous inputs, we did not measure, nor do we expect, synchrony of TRN or TC spiking to each other or within the population. TRN spiking bouts may overlap, but because our focus here is on cortical integration of transient inputs rather than synchrony, we chose not to assess fine or tight synchrony of TRN spikes.

We evaluated separation for TC neurons only, because those are the neurons that relay input to cortex.

7.) I don't understand the "separation" metric used in Figure 4. Presumably this is computed on spiking bouts of two neurons, and not temporal overlap of individual spikes from those in neurons? That should be made clear. I also can't understand whether "separation" is a bounded measure, like an index. It seems that the more positive the "separation" value is, the more distant in time two spiking bouts are. But what about the more negative "separation" values? It looks like a larger negative value indicates more overlap in spiking bouts, but suppose two spiking bouts overlapped completely. What "separation" value would they have? Presumably a very negative value, but if the "separation" value isn't bounded like an index, how are we to interpret the value that would correspond to the case of maximal spike bout overlap? Relatedly, what does "separation" value of 0 indicate? Does that mean a bout in one cell happens immediately after another? Why not just calculate this as percent overlap in time of any two spike bouts? I don't understand what this "separation" value lends us.

Yes, as you inferred separation (used here as defined in Pham and Haas, 2018) is based on the timing of bouts of spiking in each neuron; we added the word 'bouts' to the text to clarify this. It is not normalized, and not bounded.

To clarify the points you questioned and to better explain its intention, we added a sentence "Separation, measured in ms, is the time gap between spike bouts and provides an indication of whether a postsynaptic neuron might be expected to discriminate between two bouts of spiking." to the text.
